# COVID-19-Induced Sporadic Inclusion Body Myositis

**DOI:** 10.7759/cureus.30808

**Published:** 2022-10-28

**Authors:** Fazal Dalal, Hussain Dalal, Gina McNew

**Affiliations:** 1 Internal Medicine, Arkansas College of Osteopathic Medicine, Fort Smith, USA; 2 Internal Medicine, Nuvance Health, Poughkeepsie, USA; 3 Internal Medicine, Conway Regional Hospital, Conway, USA

**Keywords:** infectious myositis, sporadic inclusion body myositis, viral myositis, inclusion body myositis, covid 19

## Abstract

Sporadic inclusion body myositis (sIBM) is an autoimmune condition that is characterized by progressive weakness and muscular atrophy in the extremities. The pathophysiology is not exactly clear; however, some studies have postulated an involvement of cytotoxic T cells (CD8+), causing an autoimmune response. While the implications of COVID-19 infection are an evolving topic, myositis has been previously implicated in other viral etiologies. We discuss a case of sIBM secondary to COVID-19 in a patient with chronic myalgias bringing to light a rare implication of COVID-19 to educate the medical community.

## Introduction

Sporadic inclusion body myositis (sIBM) is an autoimmune condition that affects a variety of muscles in the body, most commonly of proximal extremities, pharyngeal muscles, and facial muscles. The clinical prodrome is usually slow-onset weakness and atrophy, which is usually triggered by an idiopathic cause [1]. Definitive diagnosis requires a muscle biopsy. Studies have shown that one of the triggers for sIBM can be a persistent viral infection. COVID-19 has been known to have lingering effects as we continue to learn more about the virus. While the implication of COVID-19 infection with myositis has been seen in a few patients, it is mostly seen as necrotizing autoimmune myositis (NAM) rather than sIBM [2]. We present a rare phenomenon of COVID-19-induced sIBM in a patient after her recent COVID-19 diagnosis.

## Case presentation

A 54-year-old female with a past medical history of asthma, hyperlipidemia on a statin, and type II diabetes mellitus presents to the primary care clinic with fatigue and myalgias, particularly of her upper and lower extremities. She had COVID-19 infection three months prior to the onset of the symptoms, and since then, she has been vaccinated with two-dose series of the COVID-19 vaccine. Statin-induced myalgias were ruled out once the patient discontinued her statin for a brief period without noticing any improvement in her symptoms; to the contrary, her symptoms worsened. Laboratory findings, including creatine kinase (CK), lactate dehydrogenase (LDH), C-reactive protein (CRP), myoglobin, aldolase, and thyroid panel, were ordered, and results are summarized in Table 1. The patient was also seen by rheumatology to rule out the autoimmune etiology of her symptoms; the results of the ordered rheumatology panel are summarized in Table 2. 

**Table 1:** Preliminary myositis laboratory tests showing trivial elevations in myositis-specific panel TSH - thyroid-stimulating hormone

Laboratory test	Laboratory value (normal)
Creatine kinase (CK)	366 (38-234 U/L)
Lactate dehydrogenase	180 (135-214 U/L)
C-reactive protein	0.61 (0.01-0.50 mg/dL)
Myoglobin	97 (25-58 ng/mL)
Aldolase	12.5 (<7.7 U/L)
TSH	1.7 (0.27-4.20 uIU/mL)

**Table 2:** Rheumatoid panel The isolated positive value for RNP is non-significant for connective tissue disease in absence of ANA positive result. Anti-CCP - anti-cyclic citrullinated peptide; ANA - antinucelar antibodies; RNP- ribonucleoprotein

Laboratory test	Laboratory value (normal)
Cortisol	11.9 (2.3-19.4 ug/dL)
Rheumatoid factor	<10 (0-14 IU/mL)
Anti-CCP	Negative (negative)
ANA	<1:80 (<1:80 reactivity)
Jo-1 antibody	<0.2 (<1.0 U)
SS-A/Ro antibody	<0.2 (<1.0 U)
SS-B/La antibody	<0.2 (<1.0 U)
Smith antibody	<0.2 (<1.0 U)
RNP antibody	3.2 (<1.0 U)
Scl-70 scleroderma antibody	<0.2 (<1.0 U)
Anti-centromere IgG antibody	<0.2 (<1.0 U)

**Figure 1:**
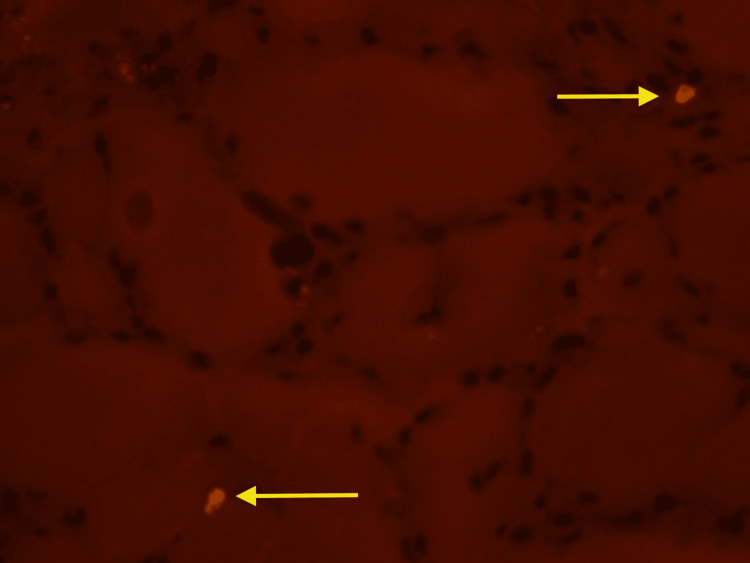
Congo red stain showing vacuoles in left calf muscle (40x magnification)

**Figure 2:**
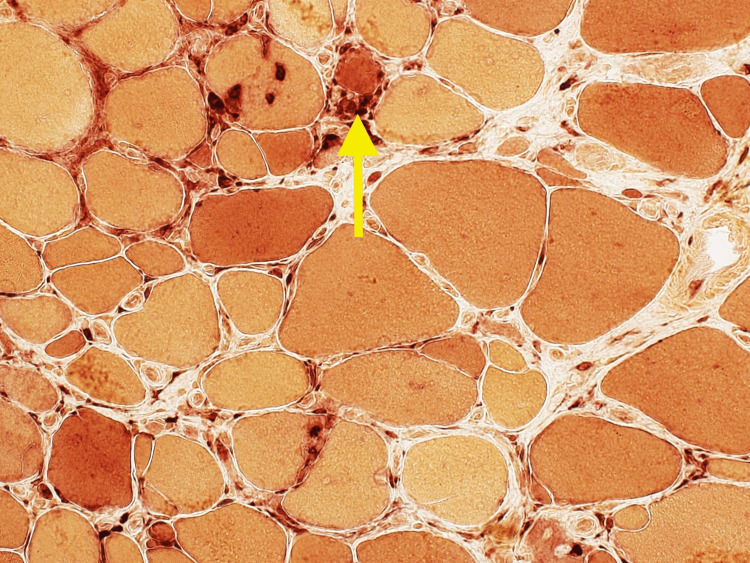
Frozen esterase stain showing left calf muscle damage with infiltrates (40x magnification)

**Figure 3:**
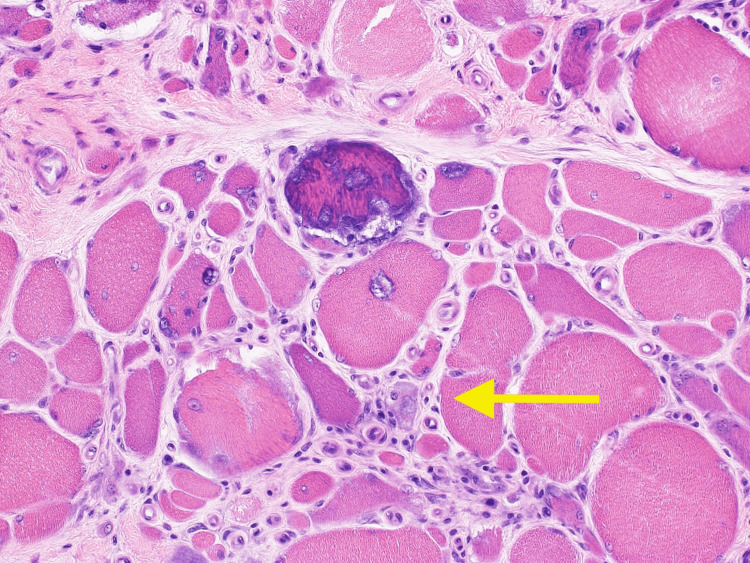
Hematoxylin and eosin stain showing endomysial inflammation and left calf muscle dystrophy (40x magnification)

**Figure 4:**
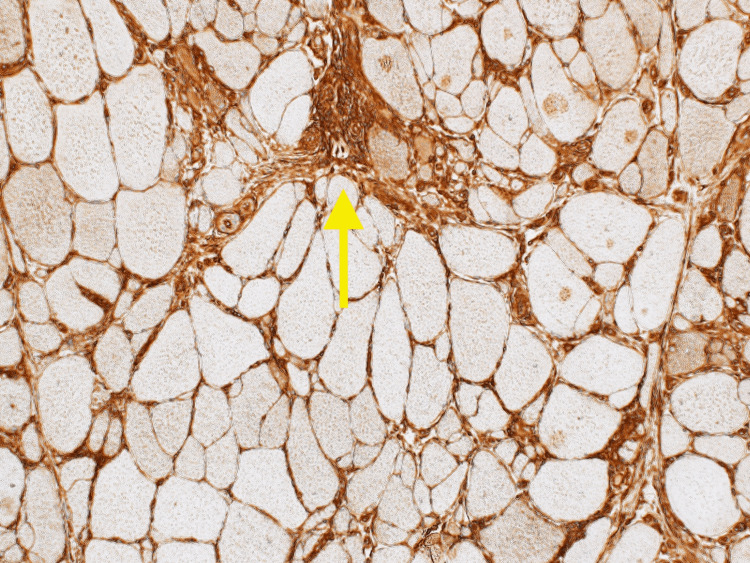
IHC stain showing CD8 cells infiltration left calf muscle cells (40x magnification) IHC - immunohistochemistry

**Figure 5:**
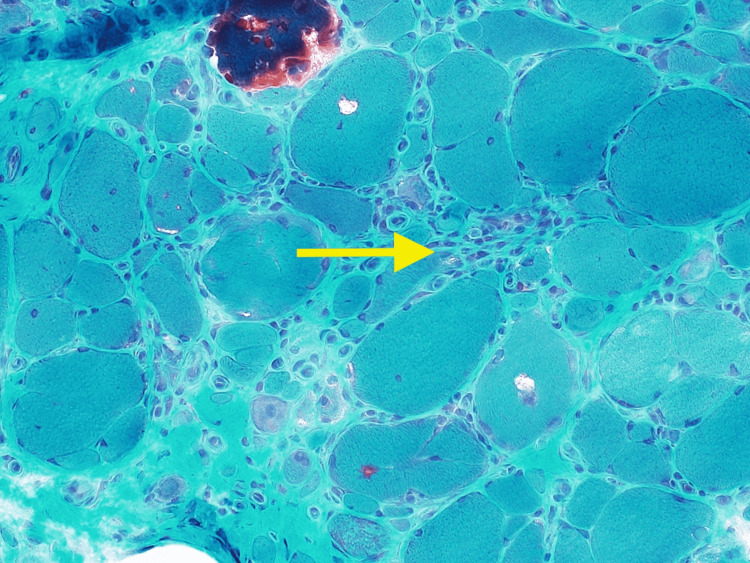
Gomori trichome stain showing rimmed vacuoles around the left calf muscle fibers (40x magnification)

As her symptoms persisted with no improvement, further diagnostic testing was undertaken. An electromyography (EMG) was conducted, which showed no evidence of any focal nerve entrapment, cervical radiculopathy, brachial plexopathy, myopathy, or generalized peripheral neuropathy. Muscle biopsy, done twice, confirmed the presence of morphologic features of an acquired inflammatory myopathy with occasional rimmed vacuole-type structures, congophilic TDP-43-p62 positive intranuclear inclusions, and amyloid-like inclusions (Figures 1-5). These features were consistent with a diagnosis of sIBM. Since there are no effective treatments for sIBM, the patient was offered physical therapy, strengthening exercises, and nonsteroidal anti-inflammatory drugs (NSAIDs) for severe pain as needed. She continues to manage her symptoms with lifestyle modifications two years after her diagnosis of sIBM. 

## Discussion

COVID-19 has certainly brought new challenges to the field of medicine as well as resulted in short and long-term sequelae. Long COVID syndrome is commonly defined as symptoms that have persisted despite recovering from COVID-19. Reasons for why these symptoms continue to persist are an area of ongoing investigation. Some of the common manifestations of long COVID are fatigue, cough, chest tightness, breathlessness, palpitations, myalgia, and lack of focus [3]. Life-threatening sequelae of post-COVID-19 infection, including stroke, myocarditis, renal failure, and pulmonary fibrosis, have been well documented [4]. Since myalgias have been associated with many viral etiologies, and differential diagnoses of myalgias are broad, we proceeded to rule out some of the most common causes initially before focusing on COVID-19 as a cause. Statin-induced myopathy was ruled out, given no resolution of her symptoms after discontinuing her statin. The trivial elevation of creatine kinase and myoglobin normalized with a repeat blood work which argued against a diagnosis of rhabdomyolysis. Once common etiologies, including autoimmune processes, were ruled out, we were able to conclude the etiology of myalgias in our patient to be COVID-19 related. 

Sporadic inclusion body myositis (sIBM) is a disease that commonly affects proximal muscles and has been associated with various etiologies. Some of the common etiologies include autoimmune, degenerative, or persistent infection. Cases of patients with HIV and human T-cell lymphotropic virus type 1 (HTLV-1) infection have been reported with myositis [5]. While the pathophysiology of retroviruses and COVID-19 is different, there seems to be a correlation between the effect of persistent viral load and sIBM. The prevalence of sIBM is relatively low at approximately 3.5 per 100,000 individuals, and that leads to delayed diagnoses of this condition [6]. The diagnosis of sIBM is made largely via clinical symptoms, but confirmation is done with a muscle biopsy. 

In our case, a muscle biopsy was imperative for a definitive diagnosis. Histologically, sIBM is characterized as lymphocytic infiltrates that invade the fibers, along with vacuolar degeneration and accumulation of amyloid proteins [7]. Different stains are used to look for macrophages and T-cells that were accumulated in the left calf muscles. Immunohistochemistry (IHC) typically shows CD8 cells infiltrating and degrading muscles, which was seen in our patient in Figure 4. Congo red stain and Gomori trichome usually show vacuoles in the muscle cells [8], which was seen in our patient as well (Figures 1 and 5). These histology findings were confirmatory of sIBM, and we proceeded with possible treatment options.

Treatment for sIBM is usually challenging owing to the nature of this slow progressive degenerative muscle disorder. The initial course of treatment is usually physical therapy, exercise, and other lifestyle modifications, which our patient has been following since her diagnosis. Although studies are ongoing on drugs that work on the transforming growth factor (TGF)-beta signaling cascade that help build some of the muscle mass lost due to sIBM [9], these studies need more sample size and analysis to prove clinical benefit. However, studies have shown that strength training has helped gain muscle strength in the least weak muscles without causing much damage to the fibers [10]. 

Our case highlights a rare implication of COVID-19, such as sIBM, that has not been reported previously. It affects the patient symptomatically for an extended period of time, and while most manifestations are non-fatal, there have been reports of respiratory failure and neurological symptoms with sIBM in some patients [11]. Fortunately, with routine follow-ups, our patient continues to gain muscle strength at every visit. With this case report, our goal was to highlight a rare form of myositis, which can be related to long COVID in a patient who has long-term manifestations of COVID-19. As the medical community continues to learn every day about COVID-19, we are sure to come across newer manifestations not previously known, which hopefully can be treated and managed clinically. 

## Conclusions

In summary, our case presents a unique clinical scenario of COVID-19-induced sporadic inclusion body myositis that has persisted for two years since COVID-19 infection. Definitive diagnosis of sIBM is usually achieved by a muscle biopsy, and treatment is supportive. We are educating the medical community about the rare evolving manifestations of COVID-19. Clinicians must have such rare manifestations of the post-COVID-19 syndrome in their differential after ruling out common etiologies of myalgias in a patient such as ours.
